# Methyl 3-[(tri­methyl­sil­yl)ethyn­yl]benzoate

**DOI:** 10.1107/S2414314626010709

**Published:** 2026-10-23

**Authors:** Melvin J. G. Lesley, Guy Crundwell, Allen G. Oliver

**Affiliations:** a Department of Chemistry Southern Connecticut State University, 501 Crescent Street New Haven CT 06515 USA; b Department of Chemistry & Biochemistry Central Connecticut State University, 1619 Stanley Street New Britain CT 06053 USA; c Molecular Structure Facility, Department of Chemistry & Biochemistry, University of Notre Dame, 149 Stepan Chemistry Hall, Notre Dame, IN 46556, USA; Purdue University, USA

**Keywords:** crystal structure, quinoxaline

## Abstract

The title compound methyl 3-[(tri­methyl­sil­yl)ethyn­yl]benzoate, C_13_H_16_O_2_Si, was synthesized *via* a Sonogashira coupling reaction using *cis*-[PdCl_2_(PPh_3_)_2_] and CuI. The two mol­ecules in the unit cell pack utilizing weak inter­molecular hydrogen-bonding inter­actions between carbon H atoms and neighboring oxygen atoms in the methyl benzoate group.

## Abstract 2

The title compound was synthesized *via* a Sonogashira coupling reaction using *cis*-[PdCl_2_ (PPh_3_)_2_] and 1 mole % CuI. The two mol­ecules in the unit cell pack utilizing weak inter­molecular hydrogen-bonding inter­actions between carbon H atoms and neighboring oxygen atoms in the methyl benzoate group.

## Graphical abstract

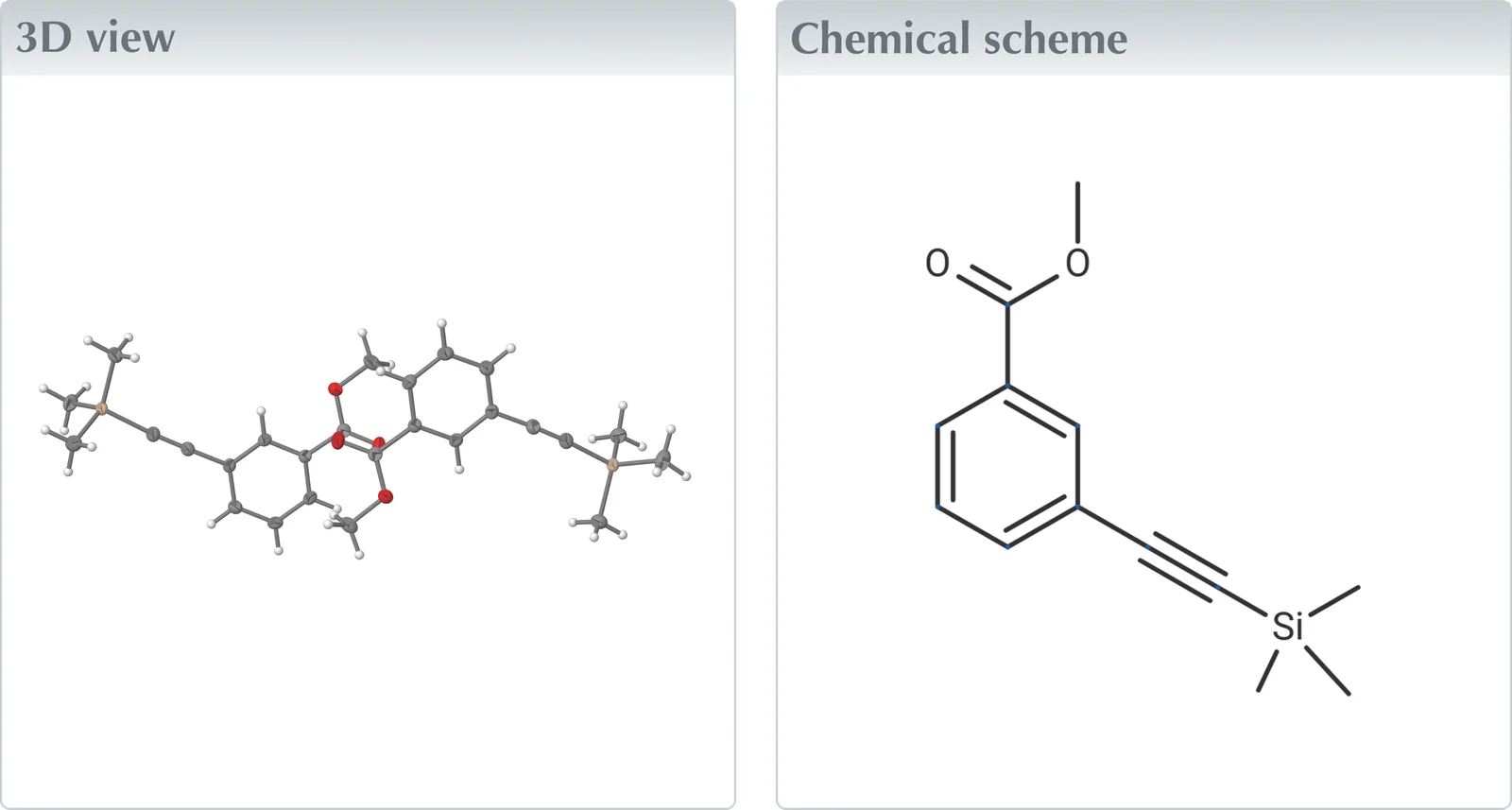

## Structure description

Coupling reactions are commonly used to develop inter­esting π-conjugated organic mol­ecules used in non-linear optical devices (Wang *et al.*, 2023 ▸). We have utilized Sonogashira cross-coupling reactions to synthesize bis­(phenyl­ethyn­yl)benzenes (Nguyen *et al.*, 1997 ▸) and report here the structure of the title compound. The structure of the related methyl 4-[(tri­methyl­sil­yl)ethyn­yl]benzoate has been published (Potts *et al.*, 2008 ▸).

**Figure 1:**
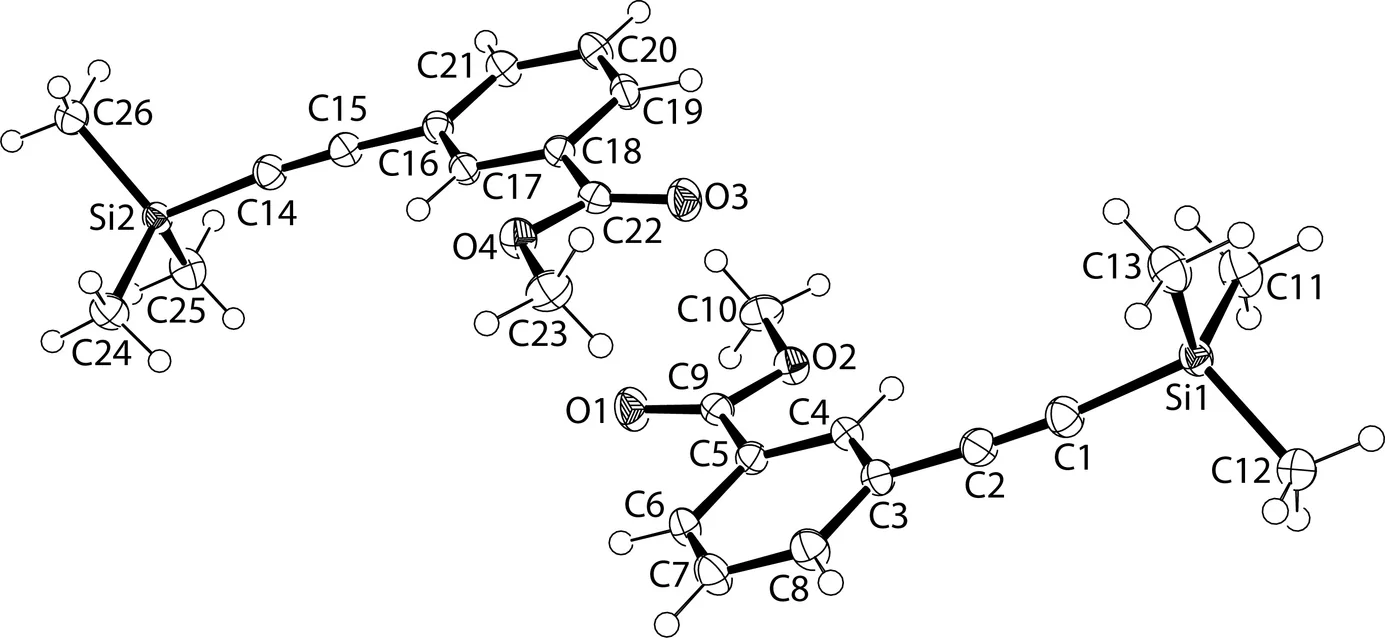
A view of the two independent mol­ecules in the title compound. Displacement ellipsoids are drawn at the 50% probability level.

**Figure 2:**
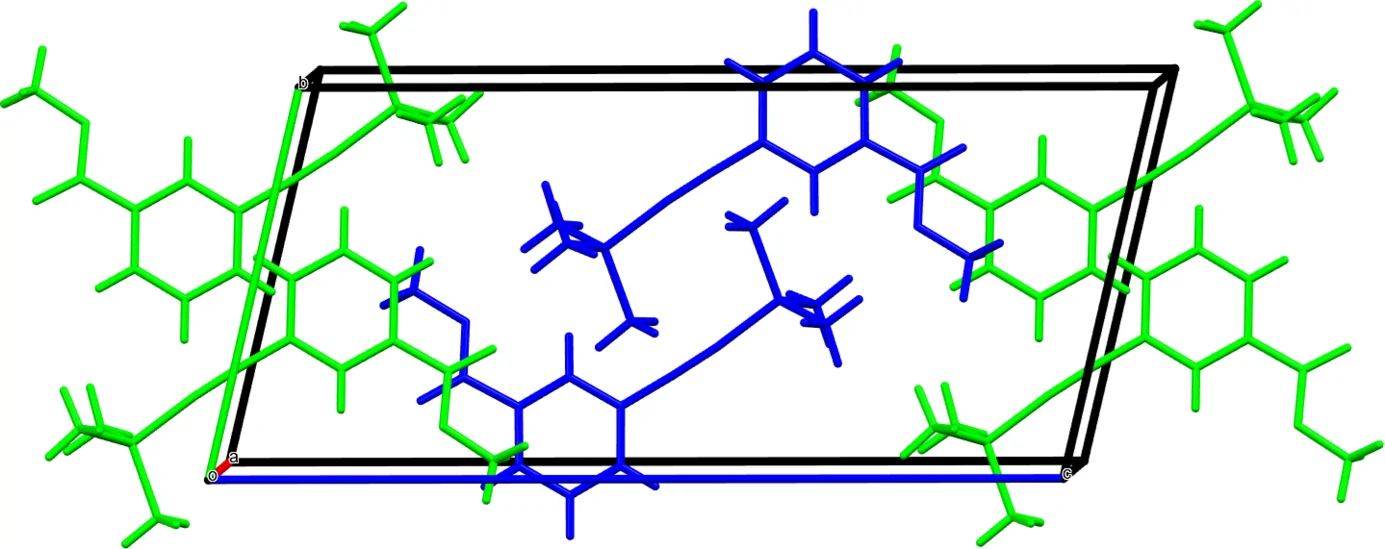
A view of unit-cell packing along [100].

**Figure 3:**
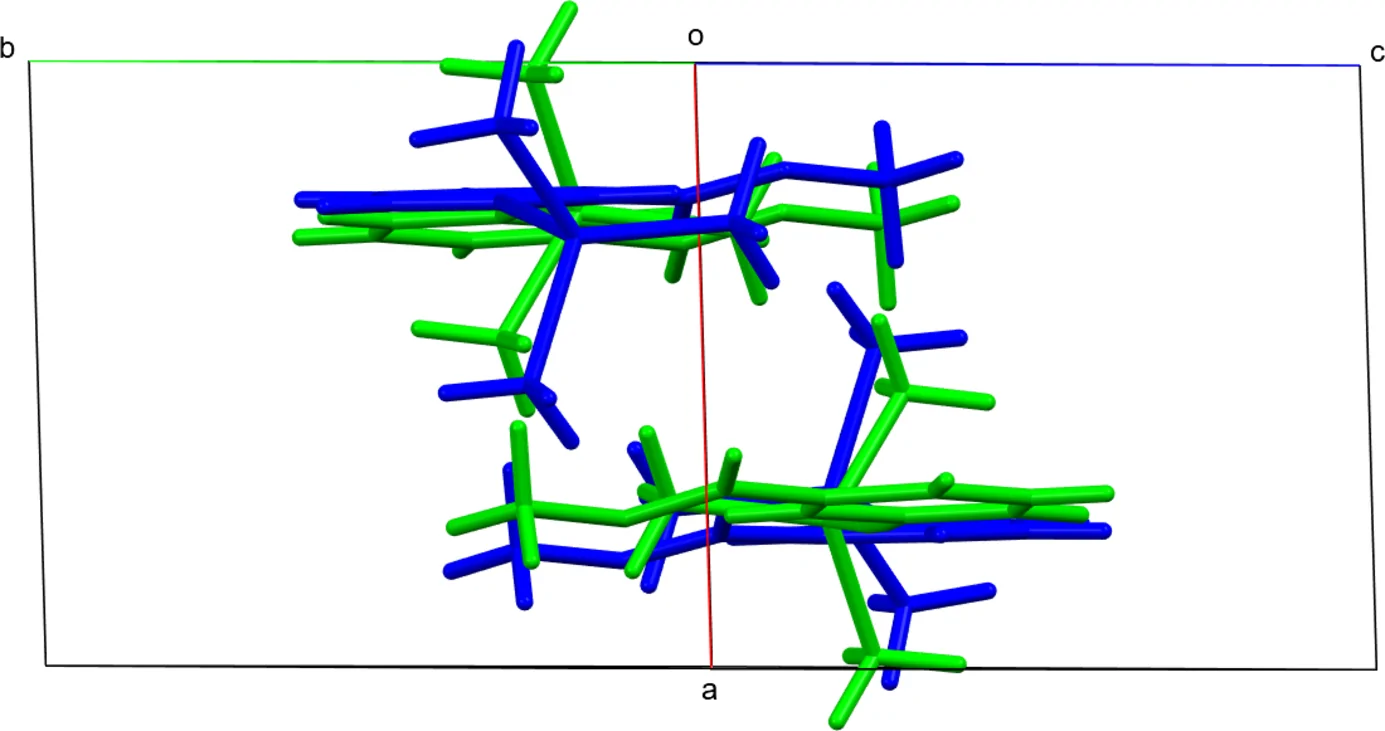
A view along [01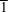] showing the pseudo-translational symmetry between the two mol­ecules in the unit cell.

There are two mol­ecules of methyl 3-[(tri­methyl­sil­yl)ethyn­yl]benzoate per asymmetric unit. Both mol­ecules are shown in Fig. 1 ▸ and a packed unit cell viewed normal to [100] is shown in Fig. 2 ▸. Both mol­ecules are basically flat with r.m.s. deviation for fitted atoms in the mol­ecules of 0.1459 and 0.1411 Å, respectively. Mol­ecules are related by pseudo-translation. A slight modulation of the two mol­ecules and a difference in rotation of the trimethyl silyl groups break exact translational symmetry that would have given A-centering, which can be seen in the view along [01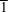] in Fig. 3 ▸.

**Figure 4:**
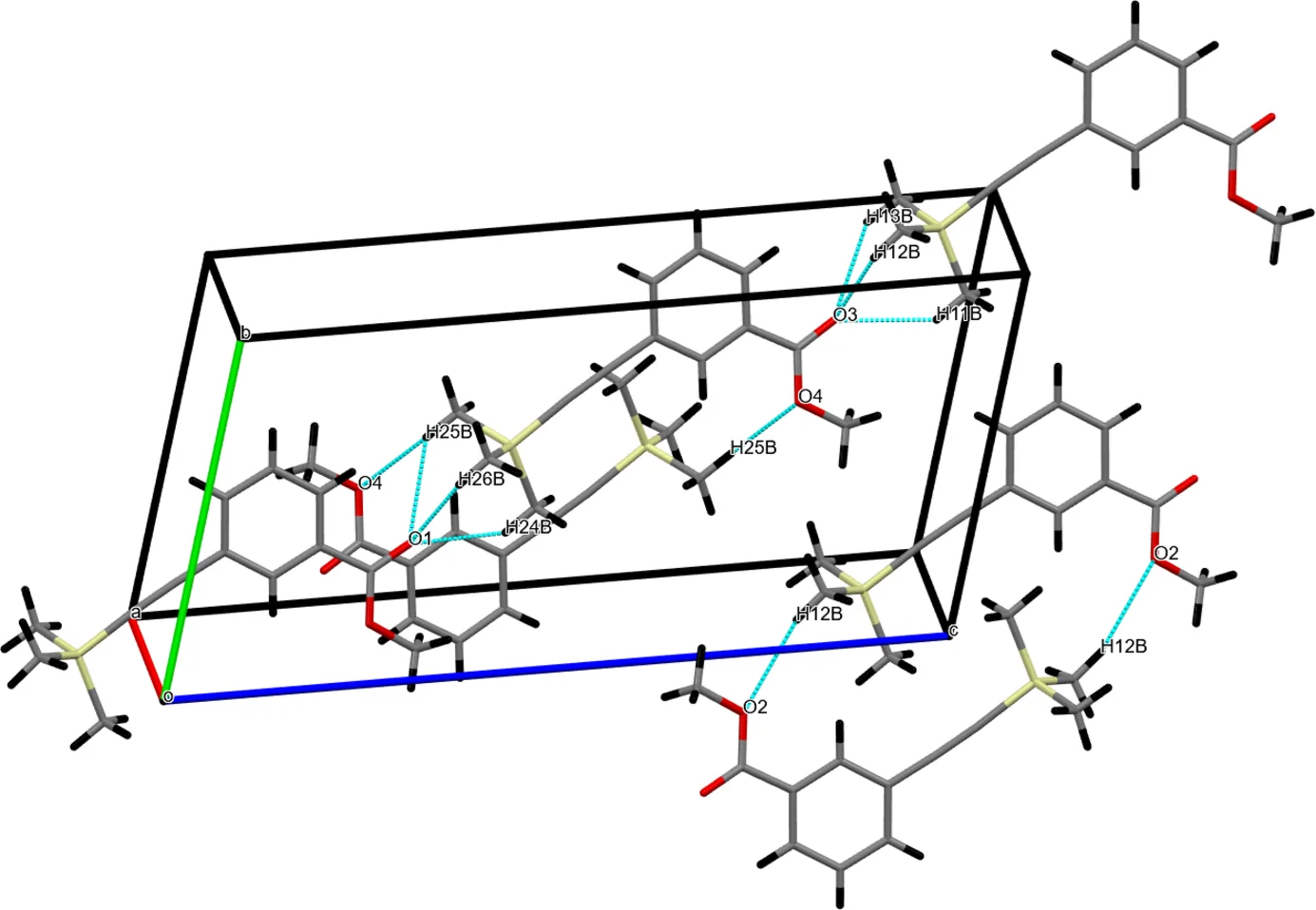
The mol­ecular packing with weak hydrogen-bonding inter­molecular inter­actions shown as blue dashed lines.

The mol­ecules pack utilizing weak hydrogen-bond inter­actions – mainly, but not exclusively, *via* tri­methyl­silyl carbon H atoms to neighboring O atoms (Table 1 ▸, Fig. 4 ▸).

## Synthesis and crystallization

Methyl-3-iodo­benzoate (19.5610 g; 74.56 mmol), 3 mol % *cis*-[PdCl_2_ (PPh_3_)_2_] (0.5830 g; 2.24 mmol), and 1 mol % CuI (0.1422 g; 0.746 mmol) were added to a 250-ml round bottom Schlenk-flask with a magnetic stir bar under N_2_. The contents of the flask were evacuated for 30 minutes and refilled with N_2_ gas, in triplicate. Degassed EtNH_2_ (200 ml) was added *via* syringe under N_2_ (*g*) (Thorand *et al.*, 1998 ▸). The reaction mixture was stirred at room temperature under a slow purge of nitro­gen. The solvent was removed *in vacuo* and the mixture was extracted with di­chloro­methane. The organic layer was isolated, dried over MgSO_4_, filtered, and reduced *in vacuo* yielding a dark-brown solution. The crude solution was purified by chromatography on neutral alumina with di­chloro­methane/hexa­nes (60:40) as eluent. Fractions from the column were reduced *in vacuo* to yield methyl 3-[(tri­methyl­sil­yl)ethyn­yl]benzoate as a red brown solid (15.500 g; 99.7%) that was used without further purification. Small crystals were selected from the *in vacuo* evaporation.

M.p: 324 K;GC–MS *m*/*z* (% relative intensity, ion): 232 (85, *M*^+^), 201 (60, *M*^+^−2CH_3_), 187 (25, *M*^+^−3CH_3_), 174 (26), 158 (40), 143 (75), 131 (32), 115 (41), 101 (33), 93 (56), 79 (59), 59 (23); FT–IR (Neat) ν (cm^−1^): 2955, 2899, 2158 (alkyne), 1728 (carbon­yl), 1599, 1578, 1481, 1436, 1423, 1288, 1250, 1209, 1105, 1090, 1074, 991, 907, 889, 844, 797, 754; ^1^H NMR (CDCl_3_, 300 MHz): δ 0.26 (*s*, 9H), 3.92 (*s*, 3H), 7.38 (*t*, *J* = 7.8 Hz, 1H), 7.64 (*d*, *J* = 7.7 Hz, 1H), 7.98 (*d*, *J* = 7.8 Hz, 1H), 8.14 (*s*, 1H); ^13^C NMR (CDCl_3_, 300 MHz): δ 0.02, 52.4, 95.5, 104.0, 123.7, 128.5, 129.6, 130.4, 133.3, 136.2, 166.5.

## Refinement

Crystal data, data collection and structure refinement details are summarized in Table 2 ▸. Three reflections (1
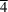
2), (1
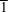
7), and (1
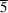
9) were flagged as problematic during initial cifcheck and were omitted from the refinement.

## Supplementary Material

Crystal structure: contains datablock(s) I. DOI: 10.1107/S2414314626010709/zl4105sup1.cif

Structure factors: contains datablock(s) I. DOI: 10.1107/S2414314626010709/zl4105Isup2.hkl

Supporting information file. DOI: 10.1107/S2414314626010709/zl4105Isup3.cml

CCDC reference: 2595593

Additional supporting information:  crystallographic information; 3D view; checkCIF report

## Figures and Tables

**Table 1:** Hydrogen-bond geometry (Å, °)

*D*—H⋯*A*	*D*—H	H⋯*A*	*D*⋯*A*	*D*—H⋯*A*
C25—H25*B*⋯O1^i^	0.98	2.82	3.5601 (13)	133
C24—H24*B*⋯O1^i^	0.98	2.52	3.3285 (13)	140
C26—H26*B*⋯O1^i^	0.98	2.74	3.4968 (13)	135
C25—H25*B*⋯O4^i^	0.98	2.99	3.7822 (13)	139
C12—H12*B*⋯O3^ii^	0.98	2.76	3.5170 (14)	135
C12—H12*B*⋯O2^iii^	0.98	3.20	3.9973 (13)	140
C12—H12*B*⋯O3^ii^	0.98	2.76	3.5170 (14)	135
C13—H13*B*⋯O2^ii^	0.98	2.94	3.8087 (13)	148
C13—H13*B*⋯O3^ii^	0.98	2.78	3.5361 (13)	134
C11—H11*B*⋯O3^ii^	0.98	2.57	3.3777 (14)	140

Symmetry codes: (i) 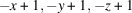; (ii) 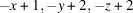; (iii) 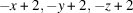.

**Table 2:** Experimental details

Crystal data	
Chemical formula	C_13_H_16_O_2_Si
*M*_r_	232.35
Crystal system, space group	Triclinic, *P* 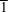
Temperature (K)	120
*a*, *b*, *c* (Å)	7.0549 (3), 9.4643 (4), 20.0892 (8)
α, β, γ (°)	77.0124 (14), 83.2760 (12), 87.6736 (13)
*V* (Å^3^)	1297.90 (9)
*Z*	4
Radiation type	Mo *K*α
μ (mm^−1^)	0.17
Crystal size (mm)	0.23 × 0.16 × 0.12

Data collection	
Diffractometer	Bruker Quest
Absorption correction	Numerical (*SADABS*; Krause *et al.*, 2015 ▸)
*T*_min_, *T*_max_	0.898, 0.918
No. of measured, independent and observed [*I* > 2σ(*I*)] reflections	65701, 6447, 5865
*R*_int_	0.042
(sin θ/λ)_max_ (Å^−1^)	0.667

Refinement	
*R*[*F*^2^ > 2σ(*F*^2^)], *wR*(*F*^2^), *S*	0.032, 0.105, 1.03
No. of reflections	6447
No. of parameters	297
H-atom treatment	H-atom parameters constrained
Δρ_max_, Δρ_min_ (e Å^−3^)	0.33, −0.20

Computer programs: *APEX5* and *SAINT* (Bruker, 2023 ▸), *SHELXT* (Sheldrick, 2015*a* ▸), *SHELXL2025/1* (Sheldrick, 2015*b* ▸), *ORTEP-3 for Windows* (Farrugia, 2012 ▸), *Mercury* (Macrae *et al.*, 2020 ▸) and *ShelXle* (Hübschle *et al.*, 2011 ▸).

## full crystallographic data

### Methyl 3-[(trimethylsilyl)ethynyl]benzoate Crystal data

**Table:**

C_13_H_16_O_2_Si	*F*(000) = 496
*M**_r_* = 232.35	*D*_x_ = 1.189 Mg m^−^^3^
Triclinic, *P*1	Melting point: 323 K
*a* = 7.0549 (3) Å	Mo *K*α radiation, λ = 0.71073 Å
*b* = 9.4643 (4) Å	Cell parameters from 9855 reflections
*c* = 20.0892 (8) Å	θ = 2.7–28.3°
α = 77.0124 (14)°	µ = 0.17 mm^−^^1^
β = 83.2760 (12)°	*T* = 120 K
γ = 87.6736 (13)°	Block, colourless
*V* = 1297.90 (9) Å^3^	0.23 × 0.16 × 0.12 mm
*Z* = 4	

### Methyl 3-[(trimethylsilyl)ethynyl]benzoate Data collection

**Table:**

Bruker Quest diffractometer	6447 independent reflections
Radiation source: micro-focus, Incoatec Iµs	5865 reflections with *I* > 2σ(*I*)
HELIOS Multi-layer monochromator	*R*_int_ = 0.042
Detector resolution: 7.41 pixels mm^-1^	θ_max_ = 28.3°, θ_min_ = 2.1°
combination of ω and φ–scans	*h* = −9→9
Absorption correction: numerical (SADABS; Krause *et al.*, 2015)	*k* = −12→12
*T*_min_ = 0.898, *T*_max_ = 0.918	*l* = −26→26
65701 measured reflections	

### Methyl 3-[(trimethylsilyl)ethynyl]benzoate Refinement

**Table:**

Refinement on *F*^2^	Primary atom site location: dual
Least-squares matrix: full	Secondary atom site location: difference Fourier map
*R*[*F*^2^ > 2σ(*F*^2^)] = 0.032	Hydrogen site location: inferred from neighbouring sites
*wR*(*F*^2^) = 0.105	H-atom parameters constrained
*S* = 1.03	*w* = 1/[σ^2^(*F*_o_^2^) + (0.0623*P*)^2^ + 0.291*P*] where *P* = (*F*_o_^2^ + 2*F*_c_^2^)/3
6447 reflections	(Δ/σ)_max_ = 0.003
297 parameters	Δρ_max_ = 0.33 e Å^−^^3^
0 restraints	Δρ_min_ = −0.20 e Å^−^^3^

### Methyl 3-[(trimethylsilyl)ethynyl]benzoate Special details

**Table:**

Geometry. All esds (except the esd in the dihedral angle between two l.s. planes) are estimated using the full covariance matrix. The cell esds are taken into account individually in the estimation of esds in distances, angles and torsion angles; correlations between esds in cell parameters are only used when they are defined by crystal symmetry. An approximate (isotropic) treatment of cell esds is used for estimating esds involving l.s. planes.
Refinement. The structure was solved by dual-space methods (Sheldrick, 2015*a*) and expanded and refined routinely (Sheldrick, 2015*b*). Hydrogen atoms on *sp*^2^ and *sp*^3^ carbons were placed at calculated positions with a C—H distance of 0.95 Å and 0.98 Å and were included in the refinement in riding motion approximation with *U*_iso_ = 1.2*U*_eq_ or 1.5*U*_eq_ of the carrier atom, respectively.

### Methyl 3-[(trimethylsilyl)ethynyl]benzoate Fractional atomic coordinates and isotropic or equivalent isotropic displacement parameters (Å^2^)

**Table:**

	*x*	*y*	*z*	*U*_iso_*/*U*_eq_	
Si1	0.71372 (4)	0.91985 (3)	1.10565 (2)	0.02029 (8)	
O1	0.73734 (12)	0.67946 (8)	0.70826 (4)	0.03097 (18)	
O2	0.82447 (11)	0.87402 (8)	0.74323 (4)	0.02402 (16)	
C4	0.77993 (13)	0.72823 (10)	0.87874 (5)	0.02011 (19)	
H4	0.785160	0.831091	0.868427	0.024*	
C5	0.78211 (13)	0.65505 (10)	0.82584 (5)	0.01945 (19)	
C1	0.74581 (15)	0.79895 (11)	1.04455 (5)	0.0253 (2)	
C16	0.25342 (13)	0.84770 (10)	0.55214 (5)	0.02016 (19)	
C17	0.26165 (13)	0.77213 (10)	0.62014 (5)	0.01965 (19)	
H17	0.252579	0.669449	0.631680	0.024*	
C18	0.28309 (13)	0.84710 (10)	0.67094 (5)	0.01896 (18)	
C6	0.77803 (15)	0.50393 (11)	0.84075 (5)	0.0231 (2)	
H6	0.779112	0.453993	0.804626	0.028*	
C7	0.77238 (16)	0.42655 (11)	0.90834 (6)	0.0271 (2)	
H7	0.772228	0.323561	0.918326	0.032*	
C8	0.76694 (16)	0.49904 (11)	0.96147 (5)	0.0260 (2)	
H8	0.761075	0.445531	1.007714	0.031*	
C9	0.77877 (14)	0.73427 (10)	0.75312 (5)	0.02109 (19)	
C2	0.75865 (15)	0.72830 (11)	1.00132 (5)	0.0233 (2)	
C3	0.77007 (14)	0.65072 (11)	0.94709 (5)	0.02120 (19)	
C11	0.73805 (18)	1.10844 (12)	1.05443 (6)	0.0319 (2)	
H11A	0.865548	1.120721	1.028827	0.048*	
H11B	0.720090	1.175952	1.085241	0.048*	
H11C	0.641367	1.128398	1.022113	0.048*	
C12	0.89790 (18)	0.87378 (13)	1.16650 (6)	0.0358 (3)	
H12A	0.873070	0.777419	1.196068	0.054*	
H12B	0.893162	0.945786	1.194935	0.054*	
H12C	1.024528	0.873874	1.140563	0.054*	
O4	0.24639 (11)	0.63216 (8)	0.75659 (4)	0.02483 (16)	
C13	0.46842 (16)	0.89055 (12)	1.15068 (6)	0.0291 (2)	
H13A	0.375464	0.916152	1.117124	0.044*	
H13B	0.446122	0.951630	1.184365	0.044*	
H13C	0.454237	0.788400	1.174308	0.044*	
O3	0.35118 (12)	0.82832 (8)	0.78586 (4)	0.02915 (17)	
C14	0.23735 (15)	0.69866 (11)	0.45654 (5)	0.0237 (2)	
C15	0.24121 (14)	0.76902 (11)	0.49946 (5)	0.0223 (2)	
C19	0.29567 (14)	0.99804 (10)	0.65403 (5)	0.02185 (19)	
H19	0.311890	1.049151	0.688567	0.026*	
C20	0.28451 (15)	1.07346 (11)	0.58690 (5)	0.0252 (2)	
H20	0.291273	1.176266	0.575681	0.030*	
C21	0.26350 (15)	0.99924 (11)	0.53602 (5)	0.0240 (2)	
H21	0.255935	1.051440	0.490098	0.029*	
C22	0.29799 (13)	0.77121 (10)	0.74346 (5)	0.02079 (19)	
C23	0.26366 (16)	0.55106 (12)	0.82587 (5)	0.0299 (2)	
H23A	0.231024	0.449607	0.829876	0.045*	
H23B	0.176497	0.592584	0.858369	0.045*	
H23C	0.395159	0.556067	0.836309	0.045*	
C24	0.27427 (16)	0.39035 (11)	0.44637 (5)	0.0270 (2)	
H24A	0.386437	0.384216	0.471473	0.040*	
H24B	0.290835	0.323181	0.415372	0.040*	
H24C	0.160154	0.364441	0.479109	0.040*	
Si2	0.24666 (4)	0.57789 (3)	0.39562 (2)	0.01924 (8)	
C25	0.45642 (16)	0.63023 (12)	0.33063 (6)	0.0299 (2)	
H25A	0.440829	0.731101	0.305991	0.045*	
H25B	0.466293	0.566774	0.297925	0.045*	
H25C	0.572722	0.620393	0.353715	0.045*	
C26	0.01931 (16)	0.60010 (12)	0.35550 (6)	0.0289 (2)	
H26A	−0.088430	0.577643	0.391462	0.043*	
H26B	0.020008	0.533995	0.324271	0.043*	
H26C	0.006678	0.700344	0.329640	0.043*	
C10	0.80769 (16)	0.96009 (12)	0.67492 (5)	0.0287 (2)	
H10A	0.893147	0.920151	0.641413	0.043*	
H10B	0.842861	1.060359	0.672555	0.043*	
H10C	0.675698	0.958132	0.664484	0.043*	

### Methyl 3-[(trimethylsilyl)ethynyl]benzoate Atomic displacement parameters (Å^2^)

**Table:**

	*U*^11^	*U*^22^	*U*^33^	*U*^12^	*U*^13^	*U*^23^
Si1	0.02528 (15)	0.01967 (14)	0.01652 (14)	−0.00021 (10)	−0.00203 (10)	−0.00542 (10)
O1	0.0445 (5)	0.0292 (4)	0.0224 (4)	0.0005 (3)	−0.0086 (3)	−0.0101 (3)
O2	0.0295 (4)	0.0210 (3)	0.0207 (3)	−0.0020 (3)	−0.0025 (3)	−0.0026 (3)
C4	0.0218 (4)	0.0177 (4)	0.0216 (5)	0.0005 (3)	−0.0030 (3)	−0.0060 (3)
C5	0.0188 (4)	0.0204 (4)	0.0197 (4)	0.0002 (3)	−0.0020 (3)	−0.0058 (3)
C1	0.0292 (5)	0.0258 (5)	0.0214 (5)	0.0002 (4)	−0.0024 (4)	−0.0064 (4)
C16	0.0205 (4)	0.0214 (4)	0.0195 (4)	−0.0006 (3)	−0.0010 (3)	−0.0069 (4)
C17	0.0203 (4)	0.0175 (4)	0.0218 (4)	−0.0004 (3)	−0.0017 (3)	−0.0060 (3)
C18	0.0182 (4)	0.0195 (4)	0.0198 (4)	0.0001 (3)	−0.0019 (3)	−0.0057 (3)
C6	0.0277 (5)	0.0205 (5)	0.0233 (5)	−0.0008 (4)	−0.0028 (4)	−0.0095 (4)
C7	0.0370 (6)	0.0173 (4)	0.0270 (5)	−0.0012 (4)	−0.0036 (4)	−0.0048 (4)
C8	0.0338 (5)	0.0218 (5)	0.0217 (5)	−0.0005 (4)	−0.0032 (4)	−0.0033 (4)
C9	0.0209 (4)	0.0212 (4)	0.0217 (4)	0.0019 (3)	−0.0017 (3)	−0.0065 (4)
C2	0.0261 (5)	0.0235 (5)	0.0203 (4)	0.0008 (4)	−0.0031 (4)	−0.0044 (4)
C3	0.0227 (4)	0.0221 (5)	0.0198 (4)	0.0008 (4)	−0.0025 (4)	−0.0070 (4)
C11	0.0436 (6)	0.0248 (5)	0.0254 (5)	−0.0056 (5)	0.0009 (4)	−0.0031 (4)
C12	0.0394 (6)	0.0374 (6)	0.0382 (6)	0.0111 (5)	−0.0174 (5)	−0.0196 (5)
O4	0.0308 (4)	0.0211 (3)	0.0215 (3)	−0.0029 (3)	−0.0037 (3)	−0.0016 (3)
C13	0.0314 (5)	0.0272 (5)	0.0287 (5)	−0.0037 (4)	0.0038 (4)	−0.0092 (4)
O3	0.0387 (4)	0.0285 (4)	0.0236 (4)	0.0009 (3)	−0.0096 (3)	−0.0100 (3)
C14	0.0258 (5)	0.0245 (5)	0.0214 (5)	−0.0001 (4)	−0.0031 (4)	−0.0059 (4)
C15	0.0240 (5)	0.0224 (5)	0.0201 (4)	−0.0005 (4)	−0.0023 (4)	−0.0039 (4)
C19	0.0241 (5)	0.0198 (4)	0.0236 (5)	−0.0016 (4)	−0.0021 (4)	−0.0089 (4)
C20	0.0314 (5)	0.0169 (4)	0.0268 (5)	−0.0021 (4)	−0.0007 (4)	−0.0046 (4)
C21	0.0288 (5)	0.0212 (5)	0.0209 (5)	−0.0005 (4)	−0.0010 (4)	−0.0034 (4)
C22	0.0197 (4)	0.0214 (4)	0.0220 (4)	0.0020 (3)	−0.0021 (3)	−0.0068 (4)
C23	0.0310 (5)	0.0299 (5)	0.0250 (5)	−0.0001 (4)	−0.0055 (4)	0.0028 (4)
C24	0.0340 (5)	0.0238 (5)	0.0219 (5)	0.0022 (4)	−0.0039 (4)	−0.0027 (4)
Si2	0.02381 (14)	0.01872 (14)	0.01593 (14)	0.00055 (10)	−0.00369 (10)	−0.00479 (10)
C25	0.0340 (6)	0.0294 (5)	0.0260 (5)	−0.0050 (4)	0.0038 (4)	−0.0082 (4)
C26	0.0314 (5)	0.0275 (5)	0.0320 (5)	0.0046 (4)	−0.0131 (4)	−0.0114 (4)
C10	0.0281 (5)	0.0289 (5)	0.0249 (5)	0.0014 (4)	−0.0033 (4)	0.0027 (4)

### Methyl 3-[(trimethylsilyl)ethynyl]benzoate Geometric parameters (Å, º)

**Table:**

Si1—C1	1.8473 (11)	C12—H12C	0.9800
Si1—C11	1.8526 (11)	O4—C22	1.3395 (12)
Si1—C13	1.8573 (11)	O4—C23	1.4468 (12)
Si1—C12	1.8598 (11)	C13—H13A	0.9800
O1—C9	1.2046 (12)	C13—H13B	0.9800
O2—C9	1.3395 (12)	C13—H13C	0.9800
O2—C10	1.4451 (12)	O3—C22	1.2070 (12)
C4—C5	1.3912 (13)	C14—C15	1.2053 (14)
C4—C3	1.3990 (13)	C14—Si2	1.8472 (11)
C4—H4	0.9500	C19—C20	1.3863 (14)
C5—C6	1.3946 (13)	C19—H19	0.9500
C5—C9	1.4870 (13)	C20—C21	1.3880 (14)
C1—C2	1.2031 (15)	C20—H20	0.9500
C16—C17	1.3990 (13)	C21—H21	0.9500
C16—C21	1.4004 (14)	C23—H23A	0.9800
C16—C15	1.4363 (13)	C23—H23B	0.9800
C17—C18	1.3922 (13)	C23—H23C	0.9800
C17—H17	0.9500	C24—Si2	1.8528 (10)
C18—C19	1.3961 (13)	C24—H24A	0.9800
C18—C22	1.4861 (13)	C24—H24B	0.9800
C6—C7	1.3873 (14)	C24—H24C	0.9800
C6—H6	0.9500	Si2—C25	1.8592 (11)
C7—C8	1.3888 (14)	Si2—C26	1.8617 (11)
C7—H7	0.9500	C25—H25A	0.9800
C8—C3	1.3997 (14)	C25—H25B	0.9800
C8—H8	0.9500	C25—H25C	0.9800
C2—C3	1.4376 (13)	C26—H26A	0.9800
C11—H11A	0.9800	C26—H26B	0.9800
C11—H11B	0.9800	C26—H26C	0.9800
C11—H11C	0.9800	C10—H10A	0.9800
C12—H12A	0.9800	C10—H10B	0.9800
C12—H12B	0.9800	C10—H10C	0.9800
			
C1—Si1—C11	107.19 (5)	H13A—C13—H13B	109.5
C1—Si1—C13	106.78 (5)	Si1—C13—H13C	109.5
C11—Si1—C13	110.35 (5)	H13A—C13—H13C	109.5
C1—Si1—C12	109.03 (5)	H13B—C13—H13C	109.5
C11—Si1—C12	111.50 (6)	C15—C14—Si2	174.44 (9)
C13—Si1—C12	111.77 (6)	C14—C15—C16	176.98 (11)
C9—O2—C10	115.16 (8)	C20—C19—C18	120.10 (9)
C5—C4—C3	120.16 (9)	C20—C19—H19	120.0
C5—C4—H4	119.9	C18—C19—H19	120.0
C3—C4—H4	119.9	C19—C20—C21	120.22 (9)
C4—C5—C6	120.03 (9)	C19—C20—H20	119.9
C4—C5—C9	121.55 (8)	C21—C20—H20	119.9
C6—C5—C9	118.34 (8)	C20—C21—C16	120.24 (9)
C2—C1—Si1	174.77 (9)	C20—C21—H21	119.9
C17—C16—C21	119.38 (9)	C16—C21—H21	119.9
C17—C16—C15	119.75 (9)	O3—C22—O4	123.65 (9)
C21—C16—C15	120.84 (9)	O3—C22—C18	123.78 (9)
C18—C17—C16	120.13 (9)	O4—C22—C18	112.57 (8)
C18—C17—H17	119.9	O4—C23—H23A	109.5
C16—C17—H17	119.9	O4—C23—H23B	109.5
C17—C18—C19	119.93 (9)	H23A—C23—H23B	109.5
C17—C18—C22	122.03 (8)	O4—C23—H23C	109.5
C19—C18—C22	118.02 (8)	H23A—C23—H23C	109.5
C7—C6—C5	119.97 (9)	H23B—C23—H23C	109.5
C7—C6—H6	120.0	Si2—C24—H24A	109.5
C5—C6—H6	120.0	Si2—C24—H24B	109.5
C6—C7—C8	120.27 (9)	H24A—C24—H24B	109.5
C6—C7—H7	119.9	Si2—C24—H24C	109.5
C8—C7—H7	119.9	H24A—C24—H24C	109.5
C7—C8—C3	120.20 (9)	H24B—C24—H24C	109.5
C7—C8—H8	119.9	C14—Si2—C24	106.75 (5)
C3—C8—H8	119.9	C14—Si2—C25	107.60 (5)
O1—C9—O2	123.72 (9)	C24—Si2—C25	111.16 (5)
O1—C9—C5	123.83 (9)	C14—Si2—C26	108.45 (5)
O2—C9—C5	112.45 (8)	C24—Si2—C26	110.90 (5)
C1—C2—C3	176.83 (11)	C25—Si2—C26	111.75 (5)
C4—C3—C8	119.34 (9)	Si2—C25—H25A	109.5
C4—C3—C2	119.43 (9)	Si2—C25—H25B	109.5
C8—C3—C2	121.21 (9)	H25A—C25—H25B	109.5
Si1—C11—H11A	109.5	Si2—C25—H25C	109.5
Si1—C11—H11B	109.5	H25A—C25—H25C	109.5
H11A—C11—H11B	109.5	H25B—C25—H25C	109.5
Si1—C11—H11C	109.5	Si2—C26—H26A	109.5
H11A—C11—H11C	109.5	Si2—C26—H26B	109.5
H11B—C11—H11C	109.5	H26A—C26—H26B	109.5
Si1—C12—H12A	109.5	Si2—C26—H26C	109.5
Si1—C12—H12B	109.5	H26A—C26—H26C	109.5
H12A—C12—H12B	109.5	H26B—C26—H26C	109.5
Si1—C12—H12C	109.5	O2—C10—H10A	109.5
H12A—C12—H12C	109.5	O2—C10—H10B	109.5
H12B—C12—H12C	109.5	H10A—C10—H10B	109.5
C22—O4—C23	115.19 (8)	O2—C10—H10C	109.5
Si1—C13—H13A	109.5	H10A—C10—H10C	109.5
Si1—C13—H13B	109.5	H10B—C10—H10C	109.5
			
C3—C4—C5—C6	−1.19 (14)	C5—C4—C3—C8	1.48 (15)
C3—C4—C5—C9	175.64 (9)	C5—C4—C3—C2	−177.01 (9)
C21—C16—C17—C18	−1.14 (14)	C7—C8—C3—C4	−0.40 (16)
C15—C16—C17—C18	176.79 (9)	C7—C8—C3—C2	178.06 (10)
C16—C17—C18—C19	0.25 (14)	C17—C18—C19—C20	0.78 (15)
C16—C17—C18—C22	−178.26 (8)	C22—C18—C19—C20	179.34 (9)
C4—C5—C6—C7	−0.20 (15)	C18—C19—C20—C21	−0.91 (16)
C9—C5—C6—C7	−177.13 (9)	C19—C20—C21—C16	0.00 (16)
C5—C6—C7—C8	1.28 (16)	C17—C16—C21—C20	1.02 (15)
C6—C7—C8—C3	−0.98 (16)	C15—C16—C21—C20	−176.89 (9)
C10—O2—C9—O1	4.31 (14)	C23—O4—C22—O3	−1.71 (14)
C10—O2—C9—C5	−174.83 (8)	C23—O4—C22—C18	178.10 (8)
C4—C5—C9—O1	−162.48 (10)	C17—C18—C22—O3	165.81 (10)
C6—C5—C9—O1	14.41 (15)	C19—C18—C22—O3	−12.72 (15)
C4—C5—C9—O2	16.67 (13)	C17—C18—C22—O4	−14.00 (13)
C6—C5—C9—O2	−166.45 (9)	C19—C18—C22—O4	167.47 (8)

### Methyl 3-[(trimethylsilyl)ethynyl]benzoate Hydrogen-bond geometry (Å, º)

**Table:**

*D*—H···*A*	*D*—H	H···*A*	*D*···*A*	*D*—H···*A*
C25—H25*B*···O1^i^	0.98	2.82	3.5601 (13)	133
C24—H24*B*···O1^i^	0.98	2.52	3.3285 (13)	140
C26—H26*B*···O1^i^	0.98	2.74	3.4968 (13)	135
C25—H25*B*···O4^i^	0.98	2.99	3.7822 (13)	139
C12—H12*B*···O3^ii^	0.98	2.76	3.5170 (14)	135
C12—H12*B*···O2^iii^	0.98	3.20	3.9973 (13)	140
C12—H12*B*···O3^ii^	0.98	2.76	3.5170 (14)	135
C13—H13*B*···O2^ii^	0.98	2.94	3.8087 (13)	148
C13—H13*B*···O3^ii^	0.98	2.78	3.5361 (13)	134
C11—H11*B*···O3^ii^	0.98	2.57	3.3777 (14)	140

Symmetry codes: (i) −*x*+1, −*y*+1, −*z*+1; (ii) −*x*+1, −*y*+2, −*z*+2; (iii) −*x*+2, −*y*+2, −*z*+2.
